# Reduced Activity in an Inpatient Liaison Psychiatry Service During the First Wave of the COVID-19 Pandemic: Comparison With 2019 Data and Characterization of the SARS-CoV-2 Positive Cohort

**DOI:** 10.3389/fpsyt.2021.619550

**Published:** 2021-02-02

**Authors:** Matthew Butler, Afraa Delvi, Fedza Mujic, Sophie Broad, Lucy Pauli, Thomas A. Pollak, Soraya Gibbs, Chun Chiang Sin Fai Lam, Marilia A. Calcia, Sotirios Posporelis

**Affiliations:** ^1^South London and Maudsley NHS Foundation Trust, London, United Kingdom; ^2^Institute of Psychiatry, Psychology and Neuroscience, London, United Kingdom; ^3^Faculty of Life Sciences and Medicine, King's College London, London, United Kingdom; ^4^Section of Women's Mental Health, IoPPN, London, United Kingdom

**Keywords:** COVID-19, SARS-CoV-2, liaison psychiatry, delirium, pandemic

## Abstract

**Background:** The COVID-19 pandemic led to changes in the way that healthcare was accessed and delivered in the United Kingdom (UK), particularly during the peak of the first lockdown period (the “first wave”) beginning in March 2020. In some patients, COVID-19 is associated with acute neuropsychiatric manifestations, and there is suggestion that there may also be longer term neuropsychiatric complications. Despite this, at the time of writing there are only emerging data on the direct effects of the COVID-19 pandemic on psychiatric care.

**Methods:** In this retrospective study we analyzed referrals to an inpatient liaison psychiatry department of a large acute teaching hospital during the first wave of covid-19 in the UK and compared this data to the same period in 2019.

**Results:** We saw a 40% reduction in the number of referrals in 2020, with an increase in the proportion of referrals for both psychosis or mania and delirium. Almost one third (28%) of referred patients tested positive for COVID-19 at some point during their admission, with 40% of these presenting with delirium as a consequence of their COVID-19 illness. Save delirium, we did not find evidence for high prevalence of new-onset acute mental illness in COVID-19 positive patients.

**Conclusion:** Our data indicate decreased clinical activity in our inpatient psychiatry liaison department during the first wave of the COVID-19 pandemic, although a relative increase in relative increase in referrals for psychosis or mania, suggesting less of a relative decrease in more severe cases of mental illness. The reasons for this are likely multifactorial, including structural changes in the NHS and patient reluctance to present to emergency departments (ED) due to infection fears and Government advice. Our data also supports the literature suggesting the high relative prevalence of delirium in COVID-19, and we support integration of psychiatry liaison teams in acute general hospital wards to optimize delirium management. Finally, consideration should be given to adequate staffing of community and crisis mental health teams to safely manage the mental health of people reluctant to visit EDs.

## Introduction

COVID-19 is the clinical syndrome caused by the novel coronavirus SARS-CoV-2. The first documented case of COVID-19 in the United Kingdom (UK) was confirmed in January 2020 (1). Since then, a number of measures have been implemented to reduce the spread of the virus, including a UK-wide lockdown on March 23rd 2020 (2). Although at the time of writing the lockdown has not been fully lifted, on May 10th 2020 the prime minister announced the introduction of the five-tiered COVID-19 alert system, heralding the end of the “first wave” of COVID-19 in the UK (3). In anticipation of a rise in COVID-19 related inpatient admissions during this first wave, acute hospitals within the National Health Service (NHS) reframed their priorities, shifting focus to safe management and treatment of COVID-19 and its complications. While justified, experts pointed out the necessity for the mental health needs of the hospitalized population to be treated with equal urgency and vigor (4).

Liaison psychiatry is a hospital-based multidisciplinary specialty which provides psychiatric input for inpatients admitted to general hospitals. Broadly, patients seen by a liaison department are those with a history of mental illness who have physical complaints, or those in whom physical illness has precipitated a change in mental state. To this end, many liaison psychiatry departments across the NHS were braced for a surge in referrals due to the effects of the COVID-19 pandemic and associated lockdown on the mental health of the population.

Many patients with COVID-19 experience mild symptoms which are confined to the respiratory tract, however COVID-19 is now understood to have broader systemic effects, including on the central nervous system (5, 6). There are data suggesting the neurotropic potential of SARS-CoV-2 in preclinical studies (7), although this has not consistently translated to clinical populations and is not generally supported by clinical studies (8). Instead, it is increasingly understood that the link between COVID-19 infections and neuropsychiatric presentations may be indirect, mediated for example through an inflammatory response (9, 10).

Neuropsychiatric consequences of previous coronavirus outbreaks showed high rates of psychiatric outcomes in infected patients. Confusion, low mood, anxiety, and impaired memory were noted in the acute stage of the illness in around a third of patients, whilst follow-up data indicated a high prevalence of post-traumatic stress disorder, depression, and anxiety (11). Currently, data are emerging on the neuropsychiatric complications associated with COVID-19 infections, which have shown similar prevalences (12, 13). There have been some data to suggest that COVID-19 is associated with new-onset psychiatric disorders such as psychosis (14, 15), however at the time of writing there is insufficient evidence to confirm this association (10, 16).

At the time of writing, an increase in psychiatric admissions in response to the COVID-19 pandemic has not been noted across Europe (17, 18). Furthermore, centrally collected data revealed a steep fall in overall Emergency Department (ED) presentations for the months following the imposition of lockdown, though details for the various patient groups remain unclear (19). Data is emerging from international studies on the specific effects of COVID-19 on psychiatric presentations; one study reported a 21% reduction in psychiatric presentations to ED in Ireland during the initial 8-week COVID-19 restrictions (20), with a similar study finding a 3-fold decrease in mental health presentations in New Zealand in comparison to previous years (21).

Despite this, there are indications that we may expect the burden on mental health services to increase in future; specifically, the longer-term effects of COVID-19 infection and the associated lockdown are yet to be established, but longer-term neuropsychiatric illnesses arising a result of COVID-19 (22, 23) are likely to add to the already significant burden that psychiatric patients and services are experiencing (24).

To our knowledge, this is the first study to report on the clinical activity of an inpatient liaison psychiatry service during the first wave of the COVID-19 pandemic and to compare it with the activity of previous periods. The study had three aims: (1) to characterize referrals made to inpatient liaison psychiatry services in terms of demographics, reasons for referral, duration of admission, diagnoses, and outcomes; (2) to compare the findings with the same period in 2019; and (3) to characterize the SARS-CoV-2 positive cohort of patients referred to liaison psychiatry, including whether COVID-19 was associated with new-onset psychiatric disorders in patients without a history of psychiatric disorders.

## Methods

### Setting

King's College Hospital (KCH) is a 950-bed capacity general acute hospital and major trauma, liver and hematology center located in Lambeth, London, one of the most ethnically diverse areas in UK. The liaison psychiatry service is a multidisciplinary team providing assessment and treatment of mental health disorders for adult (18–65 years of age) and older adult (older than 65) patients who are admitted via the emergency department (ED), or electively. The liaison psychiatry service is embedded wholly within KCH, however clinicians in the service are employed by South London and Maudsley NHS Foundation Trust (SLaM).

Inpatient referrals are received electronically through a secure NHS email address and follow a standard template. The referrals are triaged by the duty doctor and consultant during working hours and if they are accepted, the patient is reviewed face to face. At all points patients remain under the primary responsibility of the referring medical or surgical (or similar) team, with liaison psychiatry input in addition if necessary. After review by liaison psychiatry, the impression and recommendations are discussed with the referring team and the reviews are recorded on the electronic patient notes system; the patients then either remain on the liaison psychiatry caseload whilst an inpatient with ongoing input until discharge or are discharged from the caseload back to the referring team.

### Study Design

This is a retrospective inpatient electronic referrals and records review covering a 10-week period (2nd March 2020–10th May 2020, inclusive), the end of which coincides with the initial loosening of the lockdown restrictions in the UK. Data from the same 10-week period in 2019 were also extracted and anonymised and served as a comparator group. Follow-up period in 2020 was until 10th June (one month). Information was derived directly from electronic referrals. Missing information was sourced or corroborated by reviewing patient hospital records.

Data was analyzed using IBM SPSS Statistics 26. Means were compared used Student's *T*-test. Frequencies were compared using Chi-squared analysis. Alpha was set at 0.05. Z-scores were calculated using adjusted residuals from Chi-squared contingency tables and were deemed significant if ≥2.0 or ≤−2.0. Due to the descriptive and exploratory nature of the study, significance levels were not corrected for multiple comparisons.

### Data Extraction

Demographic data were obtained from the patient's records, and if not recorded, coded as Unknown. Patients were coded as either White, Asian, Black, Mixed, or Other as per the Harmonized Concepts and Questions for Social Data Sources guidance produced by the Office for National Statistics (25). Referring team details were obtained from the electronic referrals, when available. Self-referrals are those in which it is recorded that psychiatric input was explicitly requested by the patient. “Adult” patients were those aged 18–64, and “older adult” those aged >64 years. Reasons for referral were clustered in mutually agreed categories by the research team, based on the clinical question posed by the referrer.

Reasons for rejection were obtained from reviewing patient notes and were as follows: primary substance misuse issues (these were directly handled by our alcohol care team), duplicate referral, uncomplicated delirium with an obvious precipitating and treatable cause, unclear role for psychiatric input, the patient was already discharged, the patient already had outpatient (OPD) follow-up or OPD follow-up alone was appropriate, and patients with safeguarding issues without psychiatric aspects.

Legal status was coded as “informal” (referring to the default position of the patient not being subject to compulsory detention to hospital for treatment), unless at any point during the admission the patient was placed under legal frameworks of the Mental Capacity Act (more specifically a Deprivation of Liberty Safeguards, DoLS), or a Section of the Mental Health Act (1983), which mandated their detention in hospital for the period of treatment.

Characterization of patients with COVID-19 was based on hospital records at the time of obtaining a laboratory-confirmed positive SARS-CoV-2 infection using a reverse-transcriptase polymerase chain reaction assay for SARS-CoV-2 ribonucleic acid on nasopharyngeal swab or on admission if the patient was admitted for presumed COVID-19 infection. Delirium was identified based on identifying diagnoses through record review, a method which is widely used and validated (26), with high inter-rater reliability (27). First psychiatric presentation was transposed into a binary yes/no based on whether the patient had previously had one or more assessments by any clinician in any department of SLaM mental health services, based on review of their electronic notes.

## Results

### Study Population (Demographics)

**Figure 1:**
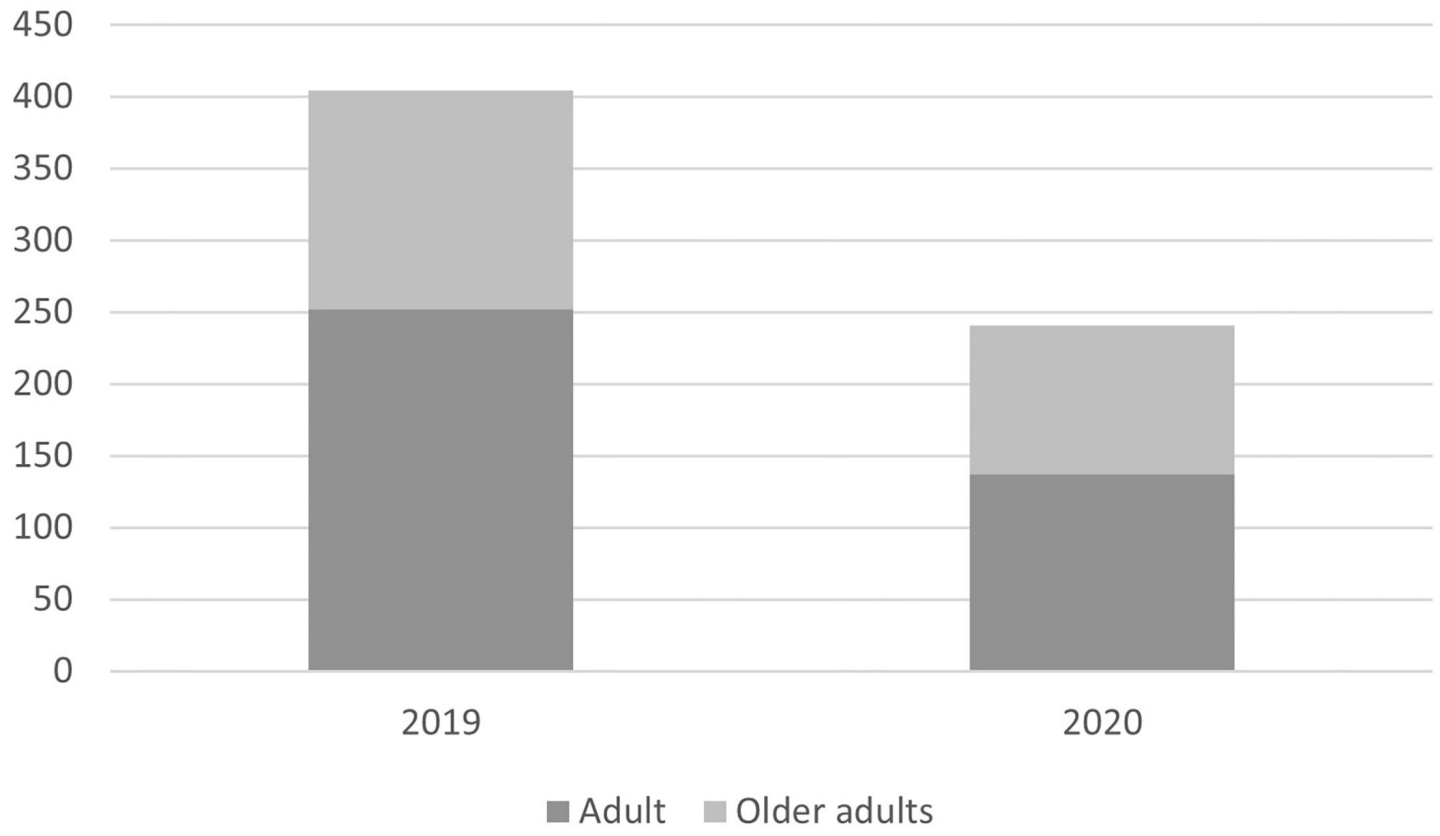
Bart chart showing decrease in number of referrals in 2020 in comparison to 2019 with total number of referrals on the y axis. The darker bars represent the proportion of referrals for adult patients, with the lighter bars representing older adults.

Between March 2nd and May 10th 2019 the KCH inpatient Liaison Psychiatry Team received a total of 404 referrals. In the same 10-week period in 2020 the referral number was 241, representing a decrease of 40.3%. The number of older adults referred decreased from 152 to 104 in 2020 in comparison to 2019 (31.6% decrease), with Adult referrals decreasing from 252 to 137 (45.6% decrease) (Figure 1). Table 1 shows demographic information of referrals by year.

**Table 1:** Demographic information of referrals by year.

	**2019**	**2020**	***p***
Mean age (years)	60.2	57.0	0.043
**Gender (%)**			
Females	188 (46.7)	114 (47.3)	n.s.
Males	216 (53.3)	127 (52.7)	n.s.
**Ethnicity (%)**			
Asian	13 (3.2)	13 (5.4)	n.s.
Black	88 (21.8)	63 (26.1)	n.s.
White	249 (61.6)	143 (59.3)	n.s.
Mixed	8 (2.0)	5 (2.1)	n.s.
Unknown	46 (11.4)	17 (7.1)	n.s.

*N.S, non-significant. P-value for age was calculated using T-test for means. Gender and ethnicity were compared with Chi-square*.

### Referral Details

Table 2 displays a breakdown of primary reasons for referral; proportionately, referrals for psychosis or mania increased by 85.1% in 2020 in comparison to 2019, and referrals for cognitive impairment increased by 71.3%, although these were only approaching significance. Although the proportions were different between the years (*X*^2^ 21.8, *d.f*. 11, *p* = 0.026), the differences were non-significant when stratified by adult and older adult. Standardized residuals (z-scores) for each referral reason were calculated for the overall data, none of which reached significance, although psychosis referrals in 2020 were approaching significance (*z* = 1.8), as were referrals for cognitive impairment (*z* = 1.6).

**Table 2:** Primary reasons for referral by year.

	**2019**			**2020**		
	**Adults**	**Older adults**	**Total (%) [*z*]**	**Adults**	**Older adults**	**Total (%) [*z*]**
Low mood	71 (28.2%)	65 (42.85)	136 (33.7%) [0.0]	39 (28.5%)	42 (40.4%)	81 (33.6%) [0.0]
Challenging behavior	23 (9.1%)	28 (18.4%)	51 (12.6%) [−0.1]	10 (7.3%)	21 (20.2%)	31 (12.9%) [0.1]
Anxiety	25 (9.9%)	11 (7.2%)	36 (8.9%) [0.4]	11 (8.0%)	7 (6.7%)	18 (7.5%) [−0.5]
Self-harm	28 (11.1%)	8 (5.3%)	36 (8.9%) [−0.1]	18 (13.1%)	4 (3.8%)	22 (9.1%) [0.1]
Psychosis or mania	27 (10.7%)	8 (5.3%)	35 (8.7%) [−1.4]	26 (19.0%)	10 (9.6%)	36 (14.9%) [1.8]
Substance misuse	21 (8.3%)	1 (0.7%)	22 (5.4%) [1.2]	5 (3.6%)	0 (0.0%)	5 (2.1%) [−1.6]
Medication advice	13 (5.2%)	7 (4.6%)	20 (5.0%) [0.6]	7 (5.1%)	1 (1.0%)	8 (3.3%) [-0.8]
Cognitive impairment	6 (2.4%)	13 (8.6%)	19 (4.7%) [−1.2]	8 (5.8%)	13 (12.5%)	21 (8.7%) [1.6]
Somatoform	16 (6.3%)	0 (0.0%)	16 (4.0%) [0.4]	7 (5.1%)	0 (0.0%)	7 (2.9%) [−0.5]
Capacity	11 (4.4%)	4 (2.6%)	15 (3.7%) [1.3]	2 (1.5%)	0 (0.0%)	2 (0.8%) [−1.7]
Other	8 (3.2%)	7 (4.6%)	15 (3.7%) [0.5]	0 (0.0%)	6 (5.8%)	6 (2.5%) [−0.7]
Psychiatric transfer	3 (1.2%)	0 (0.0%)	3 (0.7%) [−0.7]	4 (2.9%)	0 (0.0%)	4 (1.7%) [0.9]
Total	252	152	404	137	104	241

*Data were compared using Chi-square with z-scores calculated from standardized residuals*.

Table 3 shows the number of referrals to liaison psychiatry by specialty teams in the hospital. There were significantly fewer referrals from neurology in 2020 (*z* = −2.5). Referrals from acute medicine increased by 28.0% in 2020. Although not significant, referrals from ITU increased by 79.6% in 2020. Overall, the proportions of referrals by team was different (*X*^2^ 32.9, *d.f*. 10, *p* < 0.001) between the years.

**Table 3:** Referring team by speciality by year.

**Referring team**	**2019 (%) [*z*]**	**2020 (%) [*z*]**
Acute medicine	117 (39.7%) [−1.3]	121 (50.8%) [1.4]
Critical care unit	6 (2.0%) [0.0]	5 (2.1%) [0.0]
General surgery	7 (2.4%) [−0.5]	8 (3.4%) [0.5]
Geriatrics	49 (16.6%) [1.0]	28 (11.8%) [−1.1]
ITU	16 (5.4%) [−1.2]	23 (9.7%) [1.3]
Neurology	20 (6.8%) [2.2]	2 (0.8%) [−2.5]
Neurosurgery	13 (4.4%) [0.2]	9 (3.8%) [-0.3]
Orthopedics	6 (2.0%) [−1.0]	10 (4.2%) [1.1]
Other surgery	14 (4.7%) [1.3]	4 (1.7%) [−1.4]
Specialist medicine	28 (12.9%) [1.5]	16 (6.7%) [−1.7]
Trauma	9 (3.1%) [−0.8]	12 (5.0%) [0.9]

*Data were compared using Chi-square with z-scores calculated from standardized residuals*.

There were significantly more referrals rejected due to uncomplicated delirium (*z* = 3.1), as well as safeguarding concerns as opposed to mental illness being the pertinent issue (*z* = 2.0), in 2020. The overall proportions of rejection reasons by year was different (*X*^2^ 27.8, *d.f*. 6, *p* < 0.001), as per Table 4.

**Table 4:** Breakdown of SARS-CoV-2 result by liaison team, gender, and ethnicity.

	**Liaison team**		**Gender**		**Ethnicity**					
	**Adult (%)**	**Older adult (%)**	**Male (%)**	**Female (%)**	**White (%)**	**Black (%)**	**Asian (%)**	**Mixed (%)**	**Unknown (%)**	**Total**
Positive	26 (10.8)	42 (40.4)	29 (22.8)	39 (34.2)	35 (24.5)	25 (39.7)	4 (30.8)	0 (0.0)	4 (23.5)	68
Negative	76 (55.5)	56 (53.8)	74 (58.3)	58 (50.9)	87 (60.8)	28 (44.4)	6 (46.2)	4 (80.0)	7 (41.2)	132
Not tested	35 (25.5)	6 (5.8)	17 (18.9)	17 (14.9)	21 (14.7)	10 (15.9)	3 (23.1)	1 (20.0)	6 (35.3)	41
Total	137	104	127	114	143	63	13	5	17	

### Hospital Journey

Compared with 2019, in 2020 the proportion of patients referred to liaison psychiatry who had been conveyed to hospital by ambulance increased from 60.3% (*n* = 241) to 76.8% (*n* = 185), elective admissions reduced from 6.8% (*n* = 27) to 1.2% (*n* = 3), and the number of self-presentations (walk-ins) reduced from 22.3% (*n* = 89) to 14.1% (*n* = 34). Overall, the differences were significant (*X*^2^ 28.8, *d.f*. 6, *p* < 0.001). There were no differences in the proportion of patients referred to liaison psychiatry who had been admitted to ITU at some point during their admission in 2020 (*n* = 47, 19.7%) than in 2019 (*n* = 58, 14.7%) (*p* = 0.102).

There was no differences between the years in the frequency of ICD-10 diagnoses at the point of discharge. There were no significant differences in proportions of discharge destinations between the years (*X*^2^ 6.36, *d.f*. 6, *p* = 0.385). Despite this, there was a proportional increase in patients admitted to a psychiatric hospital compulsorily in 2020 (*n* = 9, 5.7% [*z* = 1.0]) in comparison to 2020 (*n* = 9, 3.2% [*z* = −0.7]), although a proportional decrease in patients transferred to a psychiatric hospital voluntarily in 2020 (*n* = 2, 1.3% [*z* = −1.0]) in comparison to 2019 (*n* = 9, 3.2% [*z* = 0.7]).

### The COVID-19 Cohort

Regarding SARS-CoV-2 status of the accepted referrals, 54.8% (*n* = 132) of patients tested negative, 28.2% (*n* = 68) tested positive while 17.0% (*n* = 41) did not have a recorded test. Table 4 shows the breakdown of test results by liaison team, gender, and ethnicity.

Legal status of SARS-CoV-2 patients was different to patients without (*X*^2^ = 17.85, *d.f*. 8, *p* = 0.22). Specifically, there were more patients on DOLS (17.6%) [*z* = 2.6] in the SARS-CoV-2 positive group vs. the SARS-CoV-2 negative (5.4%) and not tested (2.4%) groups. Delirium was present in 39.7% (*n* = 27) patients at the point of COVID-19 diagnosis, and 22.0% (*n* = 15) had a delirium as their sole presenting symptom of COVID-19.

In total, *n* = 20/68 (29.4%) of SARS-CoV-2 patients had had no known past psychiatric history (i.e., no previous contact with SLaM Mental Health services prior to the current admission). Their eventual new ICD-10 diagnoses after discharge from the liaison team in this group were organic (F0) in 55.0% (*n* = 11), neurotic (F4) in 20.0% (*n* = 4), affective (F3) in 10.0% (*n* = 2), physiological (F5) in 5.0% (*n* =1), psychotic (F2) in 5.0% (*n* = 1), and no diagnosis in 5.0% (*n* = 1). One patient was diagnosed with a new-onset psychotic disorder; this was a new-onset post-natal psychosis in an otherwise asymptomatic patient.

## Discussion

To our knowledge, this is the first attempt to describe the clinical activity of a UK-based liaison psychiatry service amidst the peak of the COVID-19 pandemic and the first to compare this with the corresponding period in the previous year. Results highlight a striking drop in the number of referrals in comparison to the same period in 2019, although, proportionately, referrals for psychosis or mania and cognitive impairment increased. The increased proportion of referrals for psychosis or mania plus proportional increase in patients admitted involuntarily may reflect the continuing presentation of the more severe end of mental illness over less severe cases. As well as this, there were proportionately less referrals for assistance with capacity assessments and substance abuse.

### Pandemic-Related Changes

Whilst the reasons behind the overall decrease in referrals may be complex, it is probably at least in part representative of a decrease in the number of overall patients who presented and were admitted to hospital during the exponential phase of the pandemic. It is possible that the population at the time of the lockdown were less willing to present to hospital due to fear of potential infection, a phenomenon which has been reflected in ED attendances for mental illnesses in other countries (20, 21), in other medical specialities in the UK (28, 29), as well as during a previous coronavirus epidemic (30). Similarly, during the period of this study, official Government advice was to stay at home unless strictly necessary, which may have led to people choosing not to attend primary or secondary healthcare settings unless in a life-threatening emergency (31).

The lower proportion of patients admitted electively in our study likely reflects changes in the provision of NHS services during the pandemic. Specifically, data from the British Medical Association suggests that in April, May, and June 2020 in England there were between 1.3 and 1.5 million fewer elective admissions than would usually be expected (32). In our study in 2020, the increase in referrals from acute medicine, and decrease from specialist medical wards, echoes the changes in drafting specialist medical doctors into more general acute medical teams in response to the changing demands of the pandemic, as well as the closure of some specialist services (33).

Overall, however, we feel that the decrease in referrals to liaison psychiatry does not represent a genuine reflection of a reduced burden of mental illness; indeed, there are indications in the literature of worsening mental health at the population level (34). Instead, the reluctance of patients to present to hospital may have added burden onto community mental health services and primary care, which was not captured in this study. We suggest that alternative methods such as reviewing primary care records or self-reported information from surveys and apps may provide a more accurate representation of incidence of mental illness than acute hospital admissions or referrals to secondary mental health services (35, 36).

Our data showed that the total number of psychiatric admissions remained relatively unchanged at discharge from liaison psychiatry, although proportionally there was an increase in involuntary admissions. Early data has indicated that admission rates to psychiatric hospitals declined during the peak of the COVID-19 pandemic in Europe in comparison to the same period in the preceding year (17, 18). This may at least in part represent changes in severity of presentations as well as the structure of mental health services during the pandemic.

### Characterizing the SARS-CoV-2 Positive Cohort

In total, 28% of referrals were SARS-CoV-2 positive; a significant proportion of which had delirium as a presenting feature, some even in the absence of other acute medical manifestations. As around 40% of SARS-CoV-2 positive patients presented with delirium as part of their COVID-19 syndrome, it is likely that referrals for COVID-19 associated delirium constituted a large proportion of this increase. Although the cause of delirium is often multifactorial, it is well-established that infections and the resultant systemic inflammatory response may lead to delirium in individuals predisposed to the condition, possibly through cytokine-induced cholinergic deficiencies (37).

Such inflammatory responses have been characterized in COVID-19 (38, 39). Data so far has indicated that the incidence of delirium in COVID-19 patients ranges from 9 to 42%, with increased incidence in those with severe infection, the elderly, and those with dementia (40–43). In many cases delirium is the sole or main presenting symptom of COVID-19 (43, 44), and, due to this, new onset of delirium has been included as a reason for clinicians to screen for COVID-19 (45).

Delirium is known to lead to stepwise worsening of cognition and may precipitate the genesis of dementia (46). Therefore, cognitive consequences in COVID-19 patients with delirium may be longstanding, a hypothesis which has been supported from early follow-up data from COVID-19 patients (47) suggesting longer-term cognitive impairment post-discharge, which is in line with evidence from longer-term follow-up from the SARS epidemic (48).

Some reports suggest that COVID-19 infection is contemporaneous with *de novo* psychiatric disorders such as psychosis and catatonia (5, 14, 15), although in many cases causality cannot be ascribed with confidence (10, 16). Others have also suggested high rates of post-traumatic stress symptoms, and higher odds of anxiety and depressive symptoms in COVID-19 inpatients in comparison to controls, although this has not been consistently replicated (49–51). In many cases in the literature, where psychiatric manifestations are noted, they are new diagnoses, although this data has also not always been replicated (5, 52, 53).

Aside from delirium, we did not find strong evidence of other new-onset psychiatric disorders arising in this cohort of SARS-CoV-2 positive patients; there were four new anxiety, two new affective disorder diagnoses, and one new post-natal psychosis diagnosis in the cohort. Although we did not record pharmacological agents in this study, use of certain medication in severe COVID-19, principally steroids, may lead to iatrogenic cases of mania or psychosis (54) as was the case in previous coronavirus outbreaks (11). This area requires further exploration.

It may, however, be the case that adverse psychiatric outcomes do not arise until after patients are discharged from hospital with COVID-19, with emerging studies indicating that patients can suffer with lasting fatigue and cognitive difficulties (55), as well as depression, anxiety, and post-traumatic stress disorder (56) for weeks after discharge. We may also see a rise in cases of severe mental illness such as psychosis with a time-lag of months or years, as has been described in previous respiratory viral epidemics (57).

Finally, in our study, a larger proportion of referrals for Black & Asian patients were SARS-CoV-2 positive in comparison to White patients. Data from the current pandemic has shown that Black, Asian and minority ethnic (BAME) people have been disproportionately affected by COVID-19, with poorer reported outcomes, including higher mortality (58). These structural health inequalities are also reflected in the experience of severe mental illnesses in BAME populations (59). In both cases, these poorer outcomes likely reflect social, economic and health inequalities, a complex social and political matter that needs to be urgently addressed.

### Limitations

There are a few limitations to the current study. This is a retrospective electronic records review and as such causal conclusions should be avoided. The data is from a single site and may not thus be generalisable in other areas; particularly, our studied population may be reflective of the diversity of a large urban area, though not representative of rural clinical environments. We did not include referrals exclusively dealing with perinatal and substance misuse presentations, as these were dealt by the respective teams. The increase in specific referrals, for example delirium, may be ascribed, at least in part to organizational restructuring within the hospital as members of the specialist dementia and delirium team were redeployed to other parts of the hospital. We did not have access to hospital records of other organizations, hence there is the possibility some of the patients being recorded as new psychiatric presentations when in fact there was a past psychiatric history. We did not collect data on pharmacological management of either COVID-19 or mental illness. Data recording the specialty of referring team was incomplete, particularly from 2019 in which 119 referrals had these specific data missing. Finally, hospital policy on swabbing for SARS-CoV-2- reflecting governmental guidelines and the provision of resources was altered during the study period; as a result, cases of COVID-19, particularly those which were asymptomatic, may have been missed as they may not have had a swab during their admission.

### Conclusions

Compared with the corresponding period in 2019, in the 10-week period during the exponential phase of the first wave of the COVID-19 pandemic in the UK, the clinical activity of our inpatient liaison psychiatry team significantly decreased, in part reflecting a change in the way people accessed health services in the UK. We saw a proportional rise in cases of cognitive impairment, which is in part explained by high rates of COVID-19 delirium. There were also proportional rises in cases of psychosis or mania, as well as patients admitted involuntarily to a psychiatric hospital at discharge, possibly suggesting less of a relative decrease in the more severe presentations. Although our results do not support the notion of consistent acute psychiatric complications of COVID-19 aside from delirium, as indicated by the low numbers of new-onset conditions, we should be mindful of potential longer-term sequelae. We would hope that adequate provision for psychiatric patients is kept at the forefront of any policy changes in response to the longer-term effects of the COVID-19 pandemic, which is ongoing at the time of writing.

## Data Availability Statement

The original contributions presented in the study are included in the article/supplementary material, further inquiries can be directed to the corresponding author/s.

## Ethics Statement

Ethical review and approval was not required for the study on human participants in accordance with the local legislation and institutional requirements. Written informed consent for participation was not required for this study in accordance with the national legislation and the institutional requirements.

## Author Contributions

Data was collected by MB, AD, FM, SB, LP, and SG. MB drafted the manuscript, performed the statistical analyses, and oversaw submission and modifications of the manuscript. SP finalized the manuscript for submission. All authors provided input on the final draft of the manuscript.

## Conflict of Interest

The authors declare that the research was conducted in the absence of any commercial or financial relationships that could be construed as a potential conflict of interest.
